# Healthcare seeking behaviour during illness among older adults in Ghana: does food security status matter?

**DOI:** 10.1186/s12877-023-04023-9

**Published:** 2023-05-25

**Authors:** Joseph Asumah Braimah, Williams Agyemang-Duah, Daniel Amoak, Yujiro Sano, Roger Antabe, Ebenezer Dassah

**Affiliations:** 1 Department of Health and Society, University of Toronto Scarborough, Toronto, Canada; 2 Department of Geography and Planning, Queen’s University, Kingston, Canada; 3 Department of Geography and Environment, Western University, London, Canada; 4 Department of Sociology, Nipissing University, North Bay, Canada; 5 School of Public Health, Kwame Nkrumah University of Science and Technology, Kumasi, Ghana

**Keywords:** Ghana, Older adults, Healthcare seeking behaviour, Food security, Multivariable analysis

## Abstract

**Background:**

Ghana’s growing older adult population raises critical questions regarding healthcare for these older adults. At the same time, food insecurity is high among older adults in Ghana. This underscores the need to investigate the issues of food security and healthcare seeking behaviour among older adults. However, research on the association between food security status and healthcare seeking behaviour among older adults is scant in the Ghanaian context. In this study, we advance the social gerontology literature by examining the association between food security status and healthcare seeking behaviors among older adults.

**Methods:**

Using a multi-stage sampling framework, we collected data from a representative sample of older adults across three regions in Ghana. Data were analyzed using logistic regression technique. We determined the significance of the test at a probability value of 0.05 or less.

**Results:**

Over two-thirds (69%) of respondents did not seek care during their last illness. Additionally, 36% of respondents were severely food insecure, 21% were moderately food insecure, 7% were mildly food insecure, and 36% were food secure. After controlling for theoretically relevant variables, our multivariable analysis revealed a statistically significant association between food security status and healthcare seeking behaviors with older people who are food secure (OR = 1.80, p < 0.01) and mildly food insecure (OR = 1.89, p < 0.05) being more likely to seek healthcare compared with their counterparts who are food insecure.

**Conclusion:**

Our findings highlight the need for sustainable intervention programs to improve food access and health service use among older adults in Ghana and similar contexts.

## Background

According to the World Health Organization [1], p8, an older adult (or older person) is an individual who is in the state of being old. The definition of an older person can be based on several criteria, including functional assessment, chronological age, legislation, or cultural factors [1], p8. To date, no standard criteria exist to conceptualize who constitutes an older adult. Despite this, various international and country-based conceptualizations of older adults have been proposed, often using chronological age [2, 3]. For instance, the Ghana Statistical Service (GSS) [3] conceptualizes an older adult as an individual who is 60 years or over. In this study, we define an older adult as someone who is 60 years or above in line with the definition provided by the GSS. Available statistics suggest that in low-and middle-income countries (LMICs), the population of older adults increased 3.7% between 2010 and 2015 and is estimated to reach 2.9% yearly before 2050 [4]. In sub-Saharan African context, there was an increased in the population of older adults from 23 million in 1990 to 46 million in 2015 and this figure is expected to rise to 161 million by 2050 [2]. In the case of Ghana, the most recent population and housing census estimated that the population of older adults aged 60 years or above is 6.4% [5], compared with 5% in 2000 [3]. These evidences suggest that the population of older adults continues to rise in LMICs, including Ghana. The rise in life expectancy, decrease in mortality rate and prolonged fertility reduction explain the increasing trend of the aging population in LMICs [6]. The growing population of older adults has some implications for psychological wellbeing [7] and healthcare systems in LMICs. Indeed, research in LMICs has established a linkage between population aging and the demand for social and healthcare services [8], which places a burden on health resources. This is because the increasing aging population results in multiple comorbid conditions [9, 10] such as diabetes, stroke etc., [11], leading to an increase in their healthcare needs which in turn influences their healthcare seeking behaviour (HSB) [12, 13].

Defined as an action undertaken by people who are perceived to have a health problem for the purpose of health restoration, HSB further explains people’s behaviour in terms of seeking for healthcare through provided health services [14]. Statistics on HSB among older adults have been reported in various studies. In a representative national survey on factors associated with HSB of older people in Nigeria, Atchessi and colleagues indicated that 53% of older adults seek healthcare when they are sick [15]. In Southern Ethiopia, 57.9% of older adults reported visiting a health center during illness [16]. In Ghana, Agyemang-Duah et al [9] found that 85% of poor older adults enrolled in a social protection scheme sought care for their health problems. These data indicate that a significant number of older adults seek care for their health problems. As old age tends to be associated with deterioration in health status and disability [17, 18], access to appropriate healthcare is crucial for maintaining their health and wellbeing.

Beyond the prevalence of HSB, studies have established the determinants of HSB in old age [10, 13, 15, 19] to include demographic, socio-economic and health-related factors. In Germany, self-rated health, chronic diseases, and physical activities influence general practitioner visits [20]. In a survey on determinants of rural-urban differentials in healthcare utilization among the elderly in India, Banerjee established that socio-economic factors such as education and economic status determine HSB among older adults [19]. In China, household expenditure, losing a partner, having multiple chronic diseases, or poor self-rated health influence HSB among older adults [21]. In Nigeria, gender, age, employment status, education, income, and place of residence explain HSB among older adults [15]. In their study on HSB of older people in Southern Ethiopia, Falaha and colleagues [16] established that variables such as age, education, type of health facility, family support during illness, and proximity of healthcare facility significantly shape HSB in old age. In Ghana, gender [22], health insurance enrollment status [23], and financial inclusion [24] are associated with HSB among older adults. Further, in their study on understanding the role of insurance in HSB, Amegbor et al. [25] found that older adults who are enrolled in the health insurance scheme in Ghana have 17% lower likelihood of using health services from a traditional healer compared to those who are not enrolled in the scheme.

Although evidence on demographic, socio-economic, and health-related factors influencing HSB exist across different countries, what is missing in these studies is the association between food security status (FSS)-defined as the ability to access adequate, safe, and nutritious food for an active and healthy life- and HSB in old age. In their study on the prevalence and predictors of food insecurity among older people in Canada, Leroux and colleagues [26] revealed that 2.4% of older Canadians are classified as moderately or severely food insecure. Statistics further show that 35.1% of older adults in Mexico, 32.9% of older adults in South Africa, 18.6% of older adults in India, 14.3% of older adults in Russia, and 44.9% of older adults in Ghana are estimated to be food insecure [27]. This underscores the need to investigate the issues of food security and HSB in old age, especially in the Ghanaian context where the prevalence of food insecurity is reported to be high in relation to other countries. Yet, there is a dearth of current research estimating the association between FSS and HSB among older adults during illness in sub-Saharan African and Ghana in particular. What makes it worthy to study the association between FSS and HSB in old age is that food (in)security is considered an emerging public health issue and, for that matter, may have implications for policy, practice, and future research. First, understanding the association between FSS and HSB may have implications for the realization of the United Nations health-related Sustainable Development Goals. Second, the findings will help inform the development of local-based policies and/or programmes aimed at concurrently improving FSS and HSB among older adults. Third, this study contributes to ongoing discussions on the relationship between FSS and HSB in old age.

There are several pathways linking FSS to HSB. Whereas food security may promote good health, food insecurity may result in poor health outcomes. In the United States, Leung, and colleagues found that food insecurity is associated with multiple chronic conditions and lower self-rated health in old age [28]. Other studies have found that food insecurity is linked to mental health problems in the United States [29], poor-self rated health in Ethiopia [30], and psychological disorders in Ghana [31]. These dynamics are likely to influence older adults to seek healthcare for their health problems. Hence, research that examines the relationship between FSS and HSB in old age during illness in Ghana is essential and timely. Whereas association between psychological disorders [31], sleep quality [32], mental distress [33], financial services [34] and food insecurity have been previously examined quantitatively among older adults in Ghana, Amoak et al. [35] investigated the link between household food insecurity and self-rated oral health in Ghana. Further, Braimah and Rosenberg [36] qualitatively investigated food access barriers and coping strategies adopted by older adults in Ghana. The study found that barriers to access to food in old age include functional impairment and poor health, poverty, insufficient social support, poor control over household resources, policy neglect, low crop yields, and sociocultural values [36]. However, what remains unclear is the linkage between FSS and HSB during illness in old age. This quantitative study seeks to build on the previous works of Braimah and Rosenberg [36], Gyasi et al. [32], Gyasi et al. [33], Amoak et al. [35], and Gyasi et al. [31] by applying the Andersen’s Behavioural Model of health services use to understand the association between FSS and HSB among older adults during illness in Ghana. The Andersen’s Behavioural Model of health services use is mostly applied to understand HSB [37, 38]. The main argument of the Andersen’s Behavioural Model is that a person’s HSB is premised on three key dimensions: predisposing, enabling and need factors [37, 38]. The predisposing factors advance the argument that the use of health services by an individual is likely to be influenced by demographic factors (such as age, gender, marital status etc.], position within the social structure and belief in health services benefits [38]. Enabling factors are the conditions that make health services obtainable to the individual [38]. These factors include food security, income, and health insurance enrollment. Need factors are the conditions that a person or health practitioner perceives as requiring treatment [39]. The applicability of Andersen’s Behavioural Model has been demonstrated in several published works in Ghana. For instance, in their study on the effect of financial services access on health services utilization among rural older adults in Ghana, Asante et al. [40] revealed that access to financial services explains health services use. In another published Ghanaian study on HSB during times of illness, Kuuire et al. [41] found that health insurance enrollment is associated with HSB among older adults in a resource poor setting. More importantly, the use of the Andersen’s Behavioural Model offers us the opportunity to select our dependent variable (HSB), primary independent variable (FSS) and control variables (such as education, occupation, marital status, religion, place of residence and health insurance among others). We theorize that FSS significantly reduces HSB among older adults during illness in Ghana.

## Materials and methods

### Data and sample

This study used data from a cross-sectional survey conducted among older people aged 60 years and over from June to August 2019. In Ghana, older adults are defined as individuals who are 60 years old and above [3]. The data were collected in three purposively sampled regions in Ghana, namely the Greater Accra, Bono East, and Upper West Regions with the assistance of research assistants. We purposively selected these regions because they fall within the three main ecological zones of Ghana [42]. For instance, the Greater Accra, Bono East (which was carved out of the old Brong Ahafo Region in 2019) and Upper West Region are situated in the Coastal, Forest and Savannah Zones, respectively. That said, the inclusion of these regions in this study offers us the opportunity to geographically capture diverse views of older adults about their HSB to inform robust and comprehensive geriatric health policy decisions. This is also consistent with a previous published work that recruited older adults from three selected regions in Ghana [43]. The data were collected using a multi-stage sampling framework. Specifically, we randomly drew two districts from each region. These districts were Sissala East Municipal, Wa West District (Upper West Region), Techiman North District, Nkoranza South District (Bono East Region), Ningo-Prampram Municipal and La Nkwantanang Madina Municipal (Greater Accra Region). Unlike a previous geriatric study in Ghana that selected one district from each of the three study regions [43], our study selected two districts from each of the three regions (Greater Accra, Bono East and Upper West Regions). This was done to enhance the robustness and representativeness of the findings. Ten enumeration areas were then randomly sampled from each district. Finally, we identified and interviewed one respondent from randomly chosen households within these enumeration areas. The research assistants, drawn from three universities in Ghana received training on questionnaire administration and ethical standards relative to the research. The research assistants were fluent in English and the local dialects of their respective regions. These were done to ensure consistency in the survey administration and prevent violations of ethical standards. Standardized data collection instruments on food security, healthcare, health behaviour, and access to care were adapted from the Ghana Living Standards Survey [44] and the WHO Study on Global Ageing and Adult Health. Our analytical sample includes 1,073 older adults. A detailed information about the procedure involved in arriving at the sample size has been reported elsewhere [35]. This study received ethical approval from the Queen’s University General Research Ethics Board (GGEOPL-277-19). In addition, verbal and written informed consents were received from the study participants. Additionally, legally Authorized Representatives of illiterate participants (e.g., family members) provided informed consent for the study.

### Measures

Respondents were asked whether they sought needed healthcare during their last illness. This question was adopted as our dependent variable called HSB- during last illness (0 = no; 1 = yes). The categorization of our dependent variable is consistent with a previous study in Ghana [41]. The focal independent variable was FSS, which was constructed based on the nine-item Household Food Insecurity Access Scale (HFIAS) [45]. Respondents were asked to answer questions about the frequency of occurrence of each food (in)security situation on a Likert scale (0 = rarely; 1 = sometimes; 2 = often) when they experienced each situation. Based on the combination of the responses from nine questions, respondents can be categorized into four groups, namely food secure, mildly food insecure, moderately food insecure, and severely food insecure (0 = food secure; 1 = mildly food insecure; 2 = moderately food insecure; 3 = severely food insecure). It is also critical to note that the relationship between FSS and HSB during last illness may be confounded by other factors. To account for possible confounders, we controlled for two sets of variables informed by the Andersen healthcare utilization model, including predisposing and enabling factors [46]. Specifically, we added education (0 = higher; 1 = secondary; 2 = primary; 3 = no education), occupation (0 = civil servant; 1 = agriculture; 2 = business; 3 = unemployed; 4 = other), marital status (0 = married; 1 = divorced; 2 = single; 3 = widowed), gender (0 = male; 1 = female), age (years) (0 = ≥ 80; 1 = 70–79; 2 = 60–69), region of residence (0 = Bono East; 1 = Greater Accra; 2 = Upper West), place of residence (0 = urban; 1 = rural), and religion (0 = Christian; 1 = Muslim; 2 = traditionalist; 3 = no religion) as part of predisposing factors while there were two enabling factors such as health insurance enrolment (0 = no; 1 = yes) and household wealth quintile (0 = richest; 1 = richer; 2 = middle; 3 = poorer; 4 = poorest).

### Analyses

There are two separate analyses for this study. First, we employed descriptive analysis to understand the characteristics of the analytical sample. Second, regression analysis was applied to understand the relationship between household FSS and HSB during last illness. Due to the binary nature of the dependent variable, we relied on logistic regression analysis. We applied sampling weights to account for the hierarchical nature of the data structure. Models were built sequentially. Model 1 explored the association between FSS and HSB during last illness, while Models 2 and 3 further controlled for predisposing and enabling factors, respectively. We determined the significance of the test at a probability value of 0.05 or less.

## Results

Sample characteristics of the respondents are reported in Table 1. The study revealed that 41% of the participants had no formal education, 35% were into agriculture, 56% were married, 53% were male and 58% were between 60 and 69 years of age. About 36% of the participants resided in the Bono East Region of Ghana, 51% lived in rural communities and 61% were Christians. Approximately 23% of the participants had enrolled in a health insurance scheme, and 20% were in the poorest household. Also, 31% of the participants sought healthcare during illness. Regarding the FSS, approximately 64% (severely, moderately, and mildly) were food insecure (see Table 1).

**Table 1:** Sample characteristics

	Percentage	
**Healthcare-seeking during illness**		
No	69	
Yes	31	
**Food security status**		
Severely food insecure	36	
Moderately food insecure	21	
Mildly food insecure	7	
Food secure	36	
**Education**		
Higher	16	
Secondary	14	
Primary	29	
No education	41	
**Occupation**		
Civil servant	12	
Agriculture	35	
Business	32	
Unemployed	13	
Other	7	
**Marital status**		
Married	56	
Divorced	9	
Single	7	
Widowed	28	
**Gender**		
Male	53	
Female	47	
**Age (years)**		
≥ 80	18	
70–79	24	
60–69	58	
**Region of residence**		
Bono East	36	
Greater Accra	29	
Upper West	35	
**Place of residence**		
Urban	49	
Rural	51	
**Religion**		
Christian	61	
Muslim	26	
Traditionalist	9	
No religion	4	
**Health insurance**		
No	77	
Yes	23	
**Household wealth**		
Richest	20	
Richer	20	
Middle	20	
Poorer	20	
Poorest	20	
Total	1,073	

Table 2 shows the logistic regression analysis predicting HSB during illness among older adults in Ghana. In Model 1, the study revealed that participants who were food secure were 2.87 times significantly more likely to seek healthcare during illness compared to those who were severely food insecure (OR = 2.87, p < 0.01). In Model 2, when other co-variates were added to the variable in Model 1, participants who classified themselves as food secure were 2.09 times significantly more likely to seek healthcare during illness compared to those who were severely food insecure (OR = 2.09, p < 0.01). However, the inclusion of these variables in Model 2 reduced the odds of the significant association between being food secure and seeking healthcare by 0.69. After fully adjusting for all theoretically relevant variables in Model 3, the results revealed that participants who were food secure were 1.80 times significantly more probable to seek healthcare during illness compared to those who were severely food insecure (OR = 1.80, p < 0.01). We observed that the addition of all theoretically relevant variables in Model 3 further reduced the odds of the significant association between those who are food secure in relation to their healthcare seeking by 0.29. However, the relationship between FSS and HSB during illness persisted after accounting for all theoretically relevant variables.

**Table 2:** Logistic regression analysis predicting healthcare seeking behaviour during illness

		Model 1			Model 2			Model 3		
		OR	95% CI		OR	95% CI		OR	95% CI	
**Food security status**										
Severely food insecure		1.00			1.00			1.00		
Moderately food insecure		1.32	0.89	1.95	1.14	0.73	1.78	1.08	0.68	1.71
Mildly food insecure		2.65***	1.59	4.43	2.16***	1.23	3.81	1.89**	1.06	3.40
Food secure		2.87***	2.09	3.93	2.09***	1.45	3.00	1.80***	1.23	2.61
**Education**										
Higher					1.00			1.00		
Secondary					1.15	0.66	2.03	1.35	0.75	2.43
Primary					1.25	0.73	2.13	1.71*	0.97	3.03
No education					0.69	0.38	1.23	1.19	0.63	2.24
**Occupation**										
Civil servant					1.00			1.00		
Agriculture					0.52**	0.29	0.93	0.73	0.39	1.36
Business					0.54**	0.30	0.95	0.66	0.36	1.21
Unemployed					0.36***	0.18	0.73	0.45**	0.22	0.96
Other					1.05	0.53	2.10	1.28	0.62	2.61
**Marital status**										
Married					1.00			1.00		
Divorced					0.89	0.52	1.52	1.07	0.62	1.84
Single					0.61	0.31	1.18	0.69	0.35	1.35
Widowed					1.13	0.79	1.62	1.26	0.87	1.83
**Gender**										
Male					1.00			1.00		
Female					0.77	0.55	1.08	0.75	0.53	1.06
**Age (years)**										
≥ 80					1.00			1.00		
70–79					0.95	0.59	1.53	0.99	0.61	1.61
60–69					0.99	0.63	1.55	1.06	0.67	1.68
**Region of residence**										
Bono East					1.00			1.00		
Greater Accra					0.62**	0.40	0.96	0.73	0.46	1.16
Upper West					1.22	0.77	1.94	1.73**	1.06	2.84
**Place of residence**										
Urban					1.00			1.00		
Rural					0.32***	0.23	0.44	0.37***	0.27	0.53
**Religion**										
Christian					1.00			1.00		
Muslim					0.89	0.61	1.29	0.95	0.64	1.41
Traditionalist					0.22***	0.09	0.50	0.22***	0.09	0.52
No religion					0.22***	0.07	0.68	0.24**	0.08	0.76
**Health insurance**										
No								1.00		
Yes								1.46*	0.96	2.22
**Household wealth**										
Richest								1.00		
Richer								1.66*	0.91	3.02
Middle								3.16***	1.72	5.83
Poorer								4.74***	2.57	8.73
Poorest								4.93***	2.54	9.55
F		16.51***			6.65***			6.21***		

*p < 0.1, **p < 0.05, ***p < 0.01

More importantly, other significant results in Model 3 are noted. First, having primary education significantly increases the odds of seeking healthcare during illness by 71% compared to higher education (OR = 1.71, p < 0.1). Second, being unemployed significantly reduces the odds of seeking healthcare during illness by 55% compared to being civil servant (OR = 0.45, p < 0.05). Third, residing in the Upper West Region of Ghana increases the odds of seeking healthcare during illness by 73% compared to living in the Bono East Region of Ghana (OR: 1.73, p < 0.05). Fourth, residing in rural areas significantly reduces the likelihood of seeking for healthcare during illness by 63% in relation to dwelling in urban areas (OR: 0.37, p < 0.01). Fifth, being affiliated to the traditionalist religion significantly reduces the probability of seeking healthcare during illness by 78% compared to being affiliated to the Christian religion (OR: 0.22, p < 0.01). Sixth, enrolling in a health insurance scheme significantly increases the odds of seeking healthcare during illness by 46% compared to not being enrolled in a health insurance scheme (OR = 1.46, p < 0.1). Seventh, participants who were in the poorest households were 4.93 times significantly more probable to seek healthcare during illness compared to those who were in the richest households (OR = 4.93, p < 0.01) (see Table 2).

## Discussion

The growth in the older adult population in Ghana, along with its associated increase in their healthcare needs, underscores the need for empirical evidence on the link between FSS and HSB among this population group. In this study, we examine the relationship between older people’s FSS and their HSB during illness. More than two-thirds of participants reported not utilizing a health facility during their last illness, which largely agrees with evidence from other recent studies [15, 16]. While the Government of Ghana has over the years embarked on population-specific healthcare promotion programs with the aim of achieving health equity [46, 47], this finding suggests that older adults are still disadvantaged in terms of healthcare access, which undermines efforts toward achieving the objectives of the United Nations Decade for Healthy Aging (2021–2030) and universal healthcare coverage. The improper alignment of the healthcare system in Ghana and, indeed most low- and middle-income countries to adequately respond to the healthcare needs of older people can be blamed for the observed low healthcare service utilization among this population group [17, 48]. For example, most healthcare systems in this context focus primarily on identifying and treating acute communicable diseases at the expense of non-communicable diseases which older adults suffer the greatest burden [8, 17]. In the context of this finding, we recommend integrated care for older people and the need to develop the competencies of healthcare systems and care providers to provide for the complex and chronic healthcare needs of older people [48].

We found that older people who are food secure are more likely to seek healthcare during sickness than their counterparts that are severely food insecure. This finding is consistent with previous studies that provide evidence to support the conclusion that food security status impacts patterns of healthcare service use among older adults [49, 50]. A possible reason for this relationship is that food insecure older people have competing needs, including feeding themselves and their dependents and hence, are less likely to go for care when ill [36, 50]. Also, the fear of not being able to comply with recommendations from healthcare providers can dissuade food insecure individuals from seeking healthcare when sick. Paradoxically, food insecurity was found to increase the likelihood of older adults utilizing office visits, inpatient hospital nights, and emergency departments in the United States of America [49]. Similarly, in Uganda, a longitudinal study among people living with HIV revealed that the severely food insecure were less likely to miss out on patient clinical visits [50]. Among others, the authors argue that health problems induced by high food insecurity may increase the use of health services by these people. Albeit conflicting, these findings collectively highlight the need for sustainable food access promotion strategies in the country to improve health service use. In Ghana, programs that promote food security are often politicized and favor the economically and socially powerful individuals to the detriment of older adults. Given this, retooling food access programs will be vital to achieving improved population health.

We further observed that socioeconomic and place-based factors influence the relationship between older people’s food security status and their healthcare seeking behaviours. Specifically, compared to older people with tertiary education, those with primary education are more likely to seek healthcare when sick. This finding contrasts an earlier study that revealed that education increases health knowledge and better healthcare negotiation power and service utilization [51]. It may however be possible that poor health knowledge due to low education serves as an incentive for older people with primary education to seek assistance from healthcare providers, including health information. Our results also reveal that wealth influences healthcare access with older people that are in the poorest, poorer, middle, and richer wealth quintiles, all having higher odds of seeking healthcare when sick compared to their counterparts in the richest wealth quintile. This is counterintuitive as previous studies establish a positive association between increased wealth and the likelihood of seeking healthcare [15, 52]. For instance, in Nigeria, Atchessi and colleagues [15] found a 25% (average) reduced healthcare usage among older people from low-income households when compared with those from wealthier households. In the specific context of Ghana, while research reveals a rise in non-communicable diseases, infectious diseases continue to dominate. These infectious diseases tend to affect individuals of low economic status, possibly explaining their high healthcare usage. Moreover, in spite of the high prevalence of non-communicable diseases among rich individuals [53], the healthcare system is generally designed to provide care for infectious diseases, which tend to spare rich people.

Consistent with previous studies, we found that place of residence shapes HSB of participants with older people that reside in rural areas being less likely to seek healthcare services than those in urban areas. Relatedly, older people in the Upper West Region of Ghana were more likely to seek healthcare when sick than their counterparts in Bono East Region. Similar locational differences in healthcare access were observed in Nigeria [15] and Ghana [47]. These results reflect geographical disparities in health service use in Ghana and most low- and middle-income countries and feed into broader discourse on health and healthcare access geographies. Rural areas in Ghana lack appropriate healthcare facilities and services, and older people who are often functionally limited have to traverse distances to urban areas to access healthcare [54]. Moreover, poverty is rife in rural areas, which can dissuade older people from seeking healthcare. Additionally, compared to Christians, older people that identify as traditionalists and those not affiliated to any religion are less likely to seek healthcare when sick. Religion importantly influences beliefs about illness including their causes and treatments. The low healthcare service utilisation observed among traditionalists may be because of their disbelieve in the formal healthcare system [55, 56]. It is also possible that traditionalists and individuals not affiliated to a religion do not receive the necessary support and encouragement their Christian counterparts get to seek healthcare [56].

Also, unemployed older people are less likely to seek healthcare compared to civil servants. It is important to mention that although individuals aged 60 and above are expected to retire, it is common in Ghana to see people above this age group in active service [3, 48], most of whom work on contract basis. The low health service utilization by unemployed older adults may be explained by the financial insecurity associated with being unemployed and its consequent effects on health service use. Kpessa-Whyte and Tsekpo [48] note that Ghana’s aging-related policies, which are primarily geared at improving financial security are limited and ineffective. Similar observations were made by Braimah, Rosenberg, and Kuuire [57] in understanding the lived experiences of left-behind older adults in the Upper West Region of Ghana. Our findings further highlight the importance of health insurance in promoting health service use among older people as older people enrolled in a health insurance program reported a higher likelihood of using healthcare service than their counterparts not enrolled in a scheme [58]. Health insurance imperatively eliminates healthcare cost to a large extent, which has been highlighted as an important barrier to health service use among older adults in low- and middle-income countries like Ghana [42, 59].

While this study provides valuable empirical evidence on the complex link between FSS and HSB of older adults in Ghana, it has a number of limitations that are worth noting. First, data for the study are cross sectional which limit our ability to establish causation and temporal trends regarding the relationship between FSS and HSB of older people. Studies drawing on longitudinal data will be useful to ascertaining the causal link between FSS and HSB. Additionally, our dependent variable, HSB during illness is self-reported and subject to recall bias. Finally, we were unable to adjust for respondents’ health status, which can affect our results and calls for consideration in future research.

## Conclusions

In a context characterized by high food insecurity and marked healthcare access disparities, this study provides information on how FSS influences health service use among older people. The study results reveal low formal healthcare utilization among older people in Ghana. We also found that FSS and HSB of older people are inextricably linked. Moreover, socioeconomic factors influence HSB of older people in Ghana. This study provides a number of recommendations for policy and practice. Firstly, there is the need for effective and sustainable programs to improve the food security of older adults in Ghana. There is also the need to expand healthcare delivery in Ghana, in terms of the number of facilities available as well as the services provided, particularly geriatric care services. Finally, making healthcare affordable, especially for older people will be helpful in enhancing their use of healthcare services.

## Data Availability

The data used and/or analysed during the current study are available from the corresponding author on reasonable request.
